# *Candida* meningitis in an infant after abdominal surgery successfully treated with intrathecal and intravenous amphotericin B: A case report

**DOI:** 10.1097/MD.0000000000027205

**Published:** 2021-09-17

**Authors:** Lihua Yuan, Feng Chen, Yao Sun, Yong Zhang, Xing Ji, Bo Jin

**Affiliations:** a Department of Pharmacy, Children's Hospital of Nanjing Medical University, Nanjing, China; b Department of Neurology, Children's Hospital of Nanjing Medical University, Nanjing, China.

**Keywords:** amphotericin B, *candida* meningitis, infant, intrathecal, intravenous

## Abstract

**Rationale:**

Studies on Candida infections in the central nervous system, especially in infants and young children that did or did not have postoperative surgery, are rarely reported. Thus far, intrathecal (*i.t.*) amphotericin B (AmB) is not routinely recommended as a therapy for Candida meningitis. We report the first case of Candida meningitis in an infant who underwent abdominal surgery and was successfully treated with *i.t.* and intravenous *(i.v.)* AmB in the mainland of China.

**Patient concerns:**

Candida meningitis was confirmed by culture and immunoserological tests in a 1-day-old girl after surgery. She was treated with fluconazole for 1 month, but the patient's symptoms showed no improvement.

**Diagnoses:**

After surgery, the infant started having recurrent attacks of fever, and laboratory tests of the cerebrospinal fluid (CSF) revealed antigens of *Candida tropicalis*. CSF tests revealed a high total protein level and a low glucose level. She was diagnosed with a secondary Candida meningitis.

**Interventions:**

After azole therapy failure, intrathecal and intravenous AmB therapy were used as rescue therapies.

**Outcomes:**

After nearly 2 months of AmB treatment, all repeat CSF cultures were negative, the infant was deemed stable and was discharged home, and she continued taking voriconazole orally as an outpatient.

**Lessons:**

The combination of *i.t.* and *i.v.* administration of AmB can provide a safe and effective alternative to managing this rare but severe disease.

## 1 Introduction

Normally, commensal *Candida* species (spp.) colonize the human gastrointestinal and genitourinary tracts. However, invasive infections may occur when a person has sores or other breaks of the skin or mucosal integrity that allow the bacteria to enter the tissue, such as the subarachnoid space (SAS), where the cerebrospinal fluid (CSF) resides, leading to *Candida* meningitis. In recent years, with improvements in the survival of infants with congenital malformations or very low birth weights in China, fungi have become a common hospital-acquired pathogen. *Candida* (spp.)^[1,2]^ is now the third (∼8%) most common cause of fungal meningitis in the United States. In China, the incidence of invasive candidiasis in the 11 participating neonatal intensive care units varied from 0.06% to 2.88% of all preterm infants and from 0.47% to 23.23% in very low birth weight infants.^[3]^ However, the *Candida* meningitis is relatively rare.

In the Chinese guidelines for the management of *Cryptococcosis*, the use of intravenous (*i.v.*) Amphotericin B (AmB) with or without fluorouracil is the first line of treatment, and intrathecal (*i.t.*) AmB is not routinely recommended. However, the international guideline for candidiasis meningitis suggests that intraventricular AmB through the device will be considered only when the intraventricular devices cannot be removed (weak recommendation),^[4]^ and *i.t.* AmB was shown to be effective for fungal meningitis in some case reports.^[5]^

Herein, we report the first case of *Candida meningitis* in an infant after an abdominal surgery who was successfully treated with *i.t.* and *i.v.* AmB in the mainland of China.

## 2 Case report

### 2.1 Patient concerns

A 1-day-old, 2.9-kg Chinese female infant developed congenital intestinal atresia combined with meconium peritonitis, and surgery was performed immediately in February 2017. She received preventive antibiotic treatment because of her repeated abdominal and bloodstream infections after the abdominal surgery. In March 2017, the infant developed *Candida* meningitis, and she was treated with curative *i.v.* voriconazole therapy.

### 2.2 Diagnosis and interventions

In April 2017, the infant started having recurrent attacks of fever and responded poorly to various antibiotic treatments. In August 2017, laboratory tests for CSF revealed the presence of antigens for *Candida tropicalis*. She was treated with fluconazole for 1 month, but the patient showed no improvement. To ensure effectiveness, preemptive treatment with voriconazole was recommended based on an antifungal susceptibility test. However, the CSF findings demonstrated a high total protein level (2.228 g/L) and a low glucose (0.48 mmol/L) after 10 days of treatment.

On September 1, 2017 (Day 1), the child was referred to our hospital for further treatment. Therapy with *i.v.* voriconazole (4 mg/kg; q12 hours) was continued according to the CSF findings and previous hospital treatment. However, the plasma and CSF levels of voriconazole were < 0.1 μg/mL after a 1-week duration. The voriconazole dosage was tailored to 7 mg/kg twice daily, but the monitored levels in the patient's plasma and CSF (i.e., 0.45 ng/mL and <0.1 ng/mL, respectively) were still lower than the target therapeutic concentration.

According to the multidisciplinary consultation team, which was comprised of physicians, pharmacologists, neurologists, infectious diseases specialists and respiratory specialists, AmB was preferred for the treatment of infant's *Candida* meningitis. However, AmB has a poor ability to cross the blood-brain barrier, and the dose-dependent pharmacokinetic characteristics, including the response to *i.v.* AmB, remains uncertain in meningitis therapy since lower CSF concentrations may be obtained. The *i.t.* administration, as a complimentary treatment, could therefore be applied to improve the AmB concentration in the brain and produce clinically curative effects. Therefore, based on the infant's response and tolerability, the infant was treated with *i.t.* AmB with a starting dose of 0.025 mg, which was increased by 0.025 mg every 2 days, in combination with *i.v.* AmB with an initial dose of 0.1 mg/kg/day, followed by a daily increase of 0.25 mg/kg. Clinical outcomes and laboratory tests were closely monitored during the period of drug treatment to observe whether the infant had developed new or concerning symptoms.

After 7 days of medication (day 25), the infant had a significant improvement in the clinical manifestations, and a repeat CSF evaluation showed a high level of glucose and reduced levels of protein (Table 1). On day 57, the patient's CSF and other laboratory test findings were close to those of healthy infants, but she developed severe renal disease. Thus, the *i.v.* AmB was discontinued according to the repeat CSF analysis (glucose level, 2.62 mmol/L; protein level, 0.36 g/L; and rare White blood cells), but the *i.t.* injection was continued until all the clinical, laboratory and radiographic signs and symptoms had improved.

**Table 1:** Cerebrospinal fluid findings of patient with *candida* meningitis.

Hospital day	Nucleated cells (×10^6^/L)	White blood cells (×10^6^/L)	Protein (g/L)	Glucose (mmol/L)
01	254	254	2.87	0.55
18	556	600	2.66	0.32
25	24	34	0.57	1.83
39	13	40	0.33	2.82
46	5	18	0.36	2.62
62	16	–^∗^	0.46	2.66
78	15	–	0.32	2.75

∗ Undetectable.

### 2.3 Outcomes

After nearly 2 months of AmB treatment, all repeat CSF cultures were negative, and the cumulative doses were 10.305 mg of *i.t.* AmB and 689.425 mg of *i.v.* AmB. The infant was deemed stable and was discharged home, and she was treated with voriconazole orally as sequential therapy after discharge. In January 2018, repeat CSF findings were all normal after she was discharged home for >1 month. After 1 year of follow-up, the infant had fully recovered.

## 3 Discussion

Some studies^[6,7]^ reported that *i.t.* AmB was successfully used to treat patients with *Cryptococcosis* meningitis, but the dosage regimen was based only on clinical experiences, descriptive studies, or expert consensus without enough evidence. Reports on *i.t.* AmB in children with *Candida* meningitis are very limited. In the present case, the infant had a high risk of death with worse CSF findings after the failure of azole therapy. Intrathecal and intravenous AmB therapy were used successfully as rescue therapies.

Because of the rarity of *Candida* meningitis, as well as AmB-related toxicity, it is not easy to determine the optimal *i.t.* dose for AmB and the therapy duration for this rare disease. Additionally, there is a lack of studies on the cumulative dose for children, and previous studies have reported that the total amount of intrathecal administered was less than 15 mg.^[8]^ Previous experiences have shown that *i.v.* doses of AmB greater than 4 g can cause irreversible kidney damage in adults. In the present case, the given dose of AmB showed significant efficacy, without obvious adverse reactions, and can provide a basis for the clinical treatment of *Candida* meningitis.

Adverse reactions to the *i.t.* administration of AmB usually occurred within 24 hours following administration, the adverse reactions included headaches, fever, back pain, radicular pain, abdominal pain, and most complications occurred in the first 2 to 3 hours.^[9]^ The infant in the present case did not experience any apparent adverse effects on the nervous system associated with the *i.t.* administration of AmB. The potential reasons could be that the *i.t.* administration procedure was performed slowly by experienced physicians in our hospital, the total *i.t.* amount of AmB was less than the recommended limit, and a low-dose level of dexamethasone (1 mg) was given after the *i.t.* administration of AmB.

Acute kidney injury is a common complication of treatment with AmB. There have been several hypotheses on the relationship between AmB and nephrotoxicity, including arteriolar vasoconstriction and direct tubular injury. One study showed that a high dose level of *i.v.* AmB resulted in nephrotoxicity, and a significant increase in blood urea nitrogen was observed. A report showed that only 36% of the cohort exhibited complete renal recovery within 30 days in adult patients receiving *i.v.* AmB^[10]^; however, AmB-associated nephrotoxicity is common and reversible in the nonneonatal pediatric population.^[11]^ In the present case, renal toxicity occurred (urea nitrogen, 22.8 mmol/L) when the *i.v.* dose was gradually increased to 20 mg/day, and the cumulative dose was 500 mg after 2 months of therapy duration. Therefore, the *i.v.* AmB was discontinued, and the infant exhibited a complete recovery in 15 days.

In conclusion, to the best of our knowledge, this is the first report in mainland China describing an infant with *Candida* meningitis post abdominal surgery who was successfully treated with the combination of *i.t.* and *i.v.* administration of AmB, which provides a safe and effective alternative to managing this rare but severe disease.

## Author contributions

**Conceptualization:** Lihua Yuan, Bo Jin.

**Data curation:** Yong Zhang.

**Investigation:** Yao Sun.

**Supervision:** Bo Jin.

**Writing – original draft:** Lihua Yuan.

**Writing – review & editing:** Feng Chen, Xing Ji.
